# Intra-hepatic Abscopal Effect Following Radioembolization of Hepatic Metastases

**DOI:** 10.1007/s00270-020-02612-4

**Published:** 2020-08-17

**Authors:** Maciej Powerski, Ralph Drewes, Jazan Omari, Borna Relja, Alexey Surov, Maciej Pech

**Affiliations:** 1 Department of Radiology and Nuclear Medicine, Otto-Von-Guericke University, Leipziger Str. 44, 39120 Magdeburg, Germany; 2 2nd Department of Radiology, Medical University of Gdansk, Mariana Smoluchowskiego 17, 80-214, Gdansk, Poland

**Keywords:** Abscopal effect, Radioembolization, SIRT

## Abstract

**Purpose:**

To search for abscopal effects (AE) distant to the site of radiation after sequential Yittrium-90 (Y-90) radioembolization (RE) of liver malignancies.

**Methods and Materials:**

In this retrospective analysis, all patients treated by RE between 2007 and 2018 (*n* = 907) were screened for the following setting/conditions: sequential RE of left and right liver lobe in two sessions, liver-specific MRI (MRI1) acquired max. 10 days before or after first RE (RE1), liver-specific MRI (MRI2) acquired with a minimum time interval of 20 days after MRI1, but before second RE (RE2). No systemic tumor therapies between MRI1 and MRI2. No patients with liver cirrhosis. Metastases > 5 mm in untreated liver lobes were compared in MRI1 and MRI2 and rated as follows: same size or larger in MRI2 = no abscopal effect (NAE); > 30% shrinkage without Y-90 contamination in SPECT/CT = abscopal effect (AE).

**Results:**

Ninety six of 907 patients met aforementioned criteria. Median time-frame between RE1 and MRI2 was 34 (20–64) days. These 96 cases had 765 metastases which were evaluable (median 5(1–40) metastases per patient). Four patients could be identified with at least one shrinking metastasis of the untreated site: one patient with breast cancer (3 metastases: 0 NAE; 3 AE), one patient with prostate cancer (6 metastases: 3 NAE; 3 metastases > 30% shrinkage but possible Y-90 contamination) and two patients with shrinkage of one metastasis each but less than 30%.

**Conclusion:**

Our retrospective study documents AE after RE of liver tumors in 1 out of 96 cases, 3 other cases remain unclear.

## Introduction

Radiotherapy (RT) represents one cornerstone of the established oncological treatment regimens. Over 60% of all cancer patients receive some form of RT during their cancer treatment [1]. The general dogmatic consensus established that the efficacy of RT is exclusively limited to the induction of cancer cell death and the eradication of clonogenic survival. However, several case reports, sporadically published over the last decades, documented the phenomenon of tumor regression at distant sites following RT, suggesting that radiation therapy has a far-reaching or delayed influence on non-irradiated cells, i.e., the abscopal effect (AE). Immune mechanisms are the major contributor to the therapeutic outcome and any AE following radiation. The mode of tumor cell death through radiation is a crucial factor for immune activation—diverse phenotypes of apoptosis, necrosis, mitotic catastrophe and senescence can be observed and are reviewed elsewhere [2]. Immunogenic forms of cell deaths can convert the tumor or metastasis into an in situ vaccine by releasing damage-associated molecular patterns (DAMPs) [3–5] unleashing a cascade of recruitment, differentiation and activation of antigen-presenting cells (APC), which are responsible for priming of an anti-tumor immunity [6, 7]. The irradiation dose, fractionation regimen and the genetic thumbprint of the irradiated cells are assumed to determine the mode of (immunogenic) cell death [2]. The clinical changes, which arise distant from the irradiated site are deemed to be the result of various factors released by the cancer cells as well as the corresponding immune cells [8]. The generation of any out-of-field-response/AE depends on whether immunosuppression or immune activation prevails in the tumor microenvironment. Several immunomodulatory substances are available to overcome immune inhibition, especially immune check-point-inhibitors have shown remarkable efficacy boosting the abscopal effects in both clinical and preclinical situations [9–11].

To our knowledge only a single report of cases, however, cover the topic of radioembolization and AE with or without immunotherapy [12].

Therefore, the goal of this retrospective study was to examine the occurrence and incidence of abscopal effects after the treatment of immunotherapy-naïve patients with radioembolization due to the rising clinical importance and potential exploitation of these out-of-field-effects for the improvement of tumor radiotherapy.

## Materials and Methods

### Ethics Approval

The study was approved by the local ethics committee.

## Patients

Nine hundred and seven patients received sequential radioembolization at our department between 2007–2018 and were screened for inclusion in this retrospective study. Inclusion criteria were: (1) an existing liver MRI with adequate quality before first (MRI1) and second (MRI2) radioembolization procedure, (2) MRI1 acquisition time max. 10 days before the first radioembolization and MRI2 acquisition time min. 20 days after MRI1/first radioembolization, (3) measurable metastases in untreated liver lobe (adequate quality and sequences of liver MRI 1 & 2). Exclusion criteria were: (1) patients with hepatocellular carcinoma and liver cirrhosis, (2) immunocompromised or immunosuppressed condition, (3) chemotherapy pause less than 3 weeks before first radioembolization.

Ninety-six patients met the inclusion criteria, consented to the use of their data, and were finally included in this retrospective analysis.

## Evaluation Angiography and Radioembolization

The detailed technique is described elsewhere [13]. The evaluation, i.e., mapping angiography and estimation of lung shunting, and radioembolization (RE) were performed according to our institute’s standard protocols via a transfemoral access on a flat-panel detector angiography system (Artis Zeego, Siemens Healthcare, Erlangen, Germany). At our institution, RE is performed in two sequential sessions (right/left) with a time interval of 4–6 weeks between treatments. We inject SIR-Spheres Microspheres (Sirtex Medical Limited, Sydney, Australia) two weeks after evaluation at a dose adjusted to the patient’s liver volume.

## Evaluated Parameters

The retrospective analysis for this study was performed by two interventional radiologists with > 5 years of experience in RE treatment in consensus using the digital subtraction angiography (DSA) and the MRI1 and MRI2 images. The Technetium macro aggregated albumin (Tc-99 m-MAA) and ^90^Y Bremsstrahlung-SPECT/CT RE treatment datasets were analyzed by a nuclear medicine specialist with several years of experience (> 5 years) in hybrid imaging.

## Statistical Analysis

All data were retrospectively analyzed using SPSS (version 24.00, IBM Corporation, NY, USA). Descriptive statistical data are presented as whole numbers (n) and percentages of the study population.

## Patient Imaging and Evaluation Parameters

Every patient received at least two Gadolinium-ethoxybenzyl-diethylenetriamine pentaacetic acid (Gd-EOB-DTPA, primovist, Bayer, Leverkusen, Germany) enhanced liver MRIs–MRI1 max. 10 days before radioembolization and MRI2 min. 20 days after MRI1 /first radioembolization.

Metastases > 5 mm in untreated liver lobes were compared in MRI1 and MRI2 and rated as follows: same size or larger in MRI2 = no abscopal effect (NAE); > 30% shrinkage and no ^90^Y accumulation in Bremsstrahlung-SPECT/CT = abscopal effect (AE). Metastasis shrinkage > 30% was deemed to be AE and a partial response according to RECIST 1.1. Extrahepatic metastases were not evaluated for AE.

## Results

**Fig. 1:**
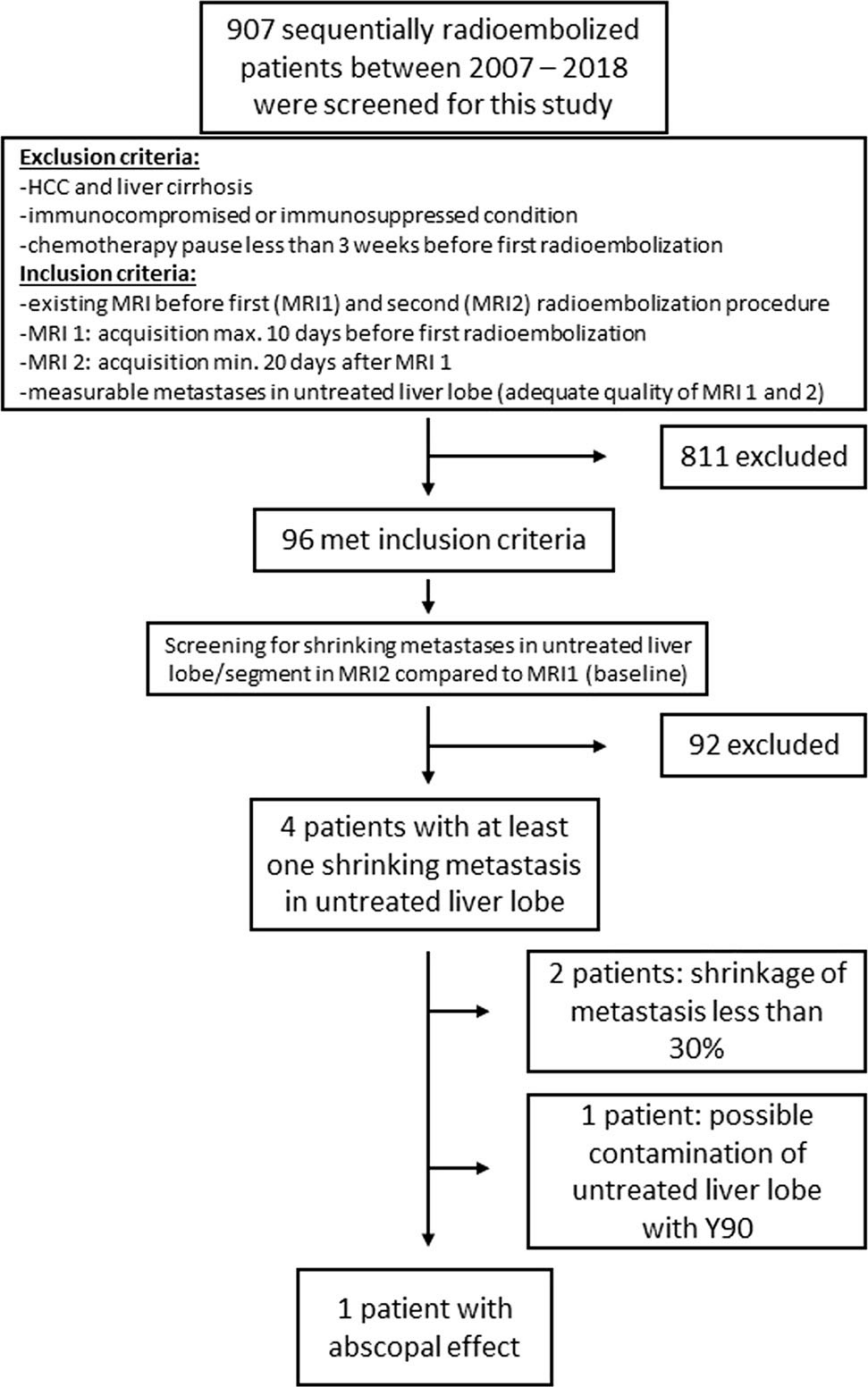
Consort diagram of inclusion and exclusion criteria showing the final study population. HCC: hepatocellular carcinoma

After the screening of 907 RE patients 96 met the inclusion criteria (Fig. 1 Consort-diagram, Table 1). The screening parameters for AE after RE and the results are summarized in Table 2.

**Table 1:** Patient characteristics

Patients screened (male/female) (*n*)	96 (56/40)	
Age (a)*	63.5 (34–84)	
Tumor entity (*n*)	CRC	55
	CCC	11
	BCA	9
	Pancreas CA	5
	Other (*n* < 5)	16
MRI 1 – first RE (days)*	1 (0–9)	
Site of first RE	Right lobe w/o Segment 4a/4b	70
	Left lobe w/o Segment 4a/4b	23
	Segments	3
Applied activity at first RE (MBq)*	1027 (340–1900)	
MRI 1–MRI 2 (days)*	34 (20–64)	
Untreated Lobe evaluated for AE in MRI 2	Right lobe w/o Segment 4a/4b	23
	Left lobe w/o Segment 4a/4b	71
	Segments	2
Untreated metastases checked for AE (n)	765	

*AE* abscopal effect,* RE* radioembolization, * median (range),* CRC* colorectal cancer,* CCC* cholangio cellular carcinoma,* BCA* breast cancer; other: prostate cancer, neuroendocrine carcinoma, lung cancer, anal cancer, cervix cancer, plasmocytoma, endometrial cancer, carcinoid tumor, pharyngeal cancer, stomach cancer, duodenal cancer

**Table 2:** Screening for abscopal effects after radioembolization

*Comparison of untreated metastases in MRI 1 (baseline) and MRI 2 (follow-up after first RE)*											
RECIST 1.0 (96 patients)		Response of lesions (765 checked for AE)									
Complete response (CR)	0	Complete response—0 metastases									
Partial response (PR)	2	Shrinkage > 30%—6 metastases (2 patients)									
Stable disease (SD)	35	Shrinkage < 30%—2 metastases (2 patients)									
Progressive disease (PD)	61	No shrinkage or progression – 757 metastases									
*Patients with PR*											
Patient A (compare Fig. 2) (female, age 43, BCA)	04/2010—initial diagnosis of bilateral breast cancer 12/2013—first detection of liver metastases (ER +, PR +, Her2neu-) Endocrine therapy with Tamoxifen und GnRH analogues Decision for radioembolization due to progressive hepatic disease 10/2015 radioembolization of the right liver lobe + S4b (1270 MBq) Abscopal effect registered (acquisition of MRI2 42 days after MRI1 and 43 days after first RE) 11/2015 radioembolization of the left liver lobe (640 MBq) 5/16 stable disease Lost in further follow-up										
	Shrinkage of metatsases (mm)	MRI1					MRI2				
		18		19		31	14		14		19
Patient B (compare Fig. 3) (male, age 65, Prostate CA)	03/1995—initial diagnosis of prostate cancer 01/2002—first detection of liver metastases chemotherapy cycles with: estramustine, docetaxel, capecitabine, imatinib – until 04/2005 06/2005—11/2006 multiple local ablations (interstitial brachytherapy) of liver metastases 11/2007 radioembolization of the right liver lobe (850 MBq) Questionable Abscopal effect registered (acquisition of MRI2 42 days after MRI1 and 43 days after first RE) 01/2008 radioembolization of the left liver lobe (400 MBq) 09/2008 deceased										
	Shrinkage of metatsases (mm)	MRI1					MRI2				
		10	20	24	28	34	10	14	24	21	27

**Fig. 2:**
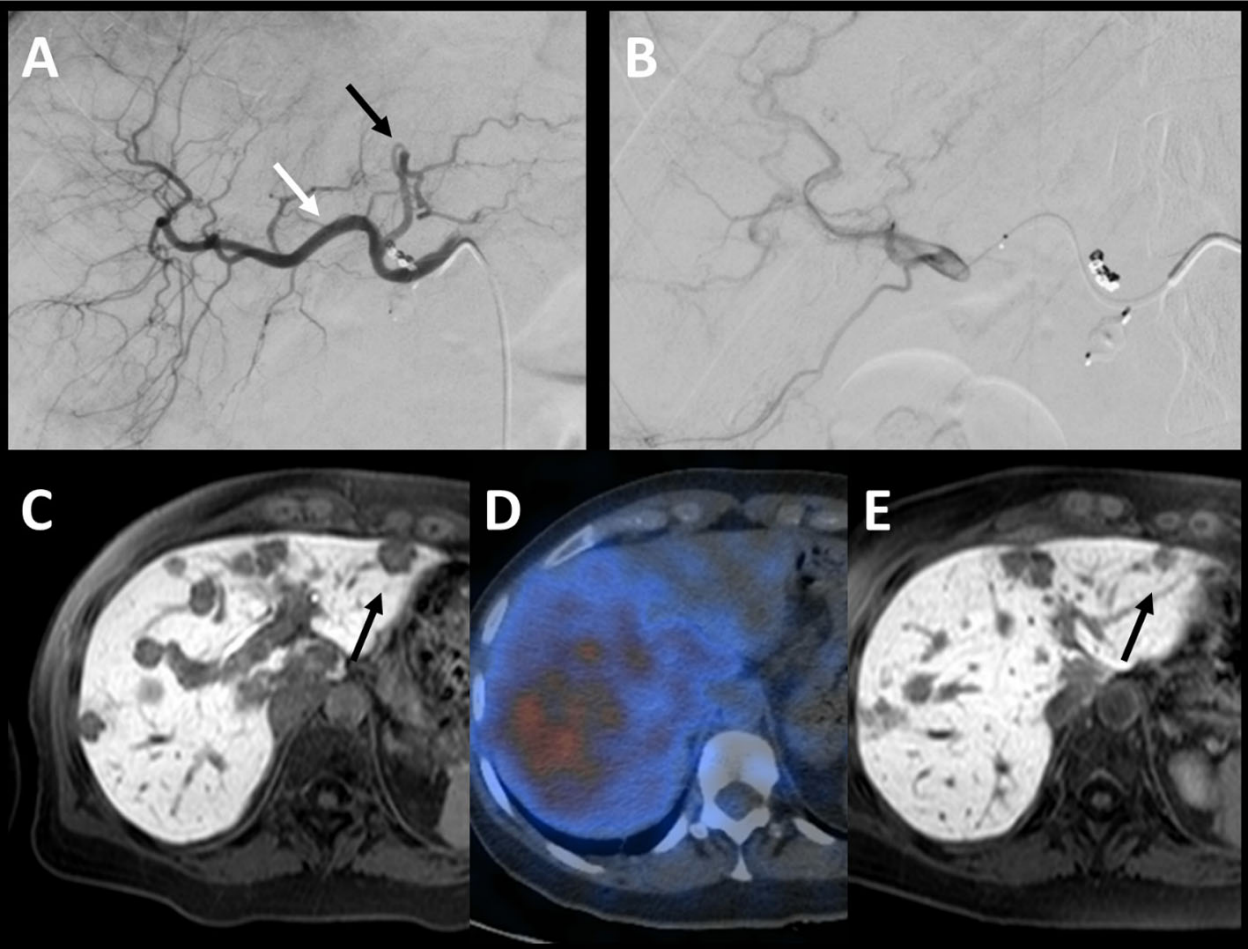
Patient A, breast cancer, A-E, certain abscopal effect: **A** white arrow demonstrates the injection of Tc-99 m-MAA /the evaluation point of segments 5,6,7,8,1 and partial 4; black arrow shows the evaluation point left and partial segment 4. **B** application of ^90^Y right (5–8, 1 and partial segment 4). **C** MRI1: T1w 20 min after administration of primovist—late enhancement phase 1 day prior to RE, black arrow shows metastasis prior to treatment of the right liver lobe. **D** Bremsstrahlung-SPECT/CT examination: no ^90^Y distribution in the left liver lobe. **E** MRI2: 42 days after the first RE—black arrow shows untreated metastasis decreased in diameter

**Fig. 3:**
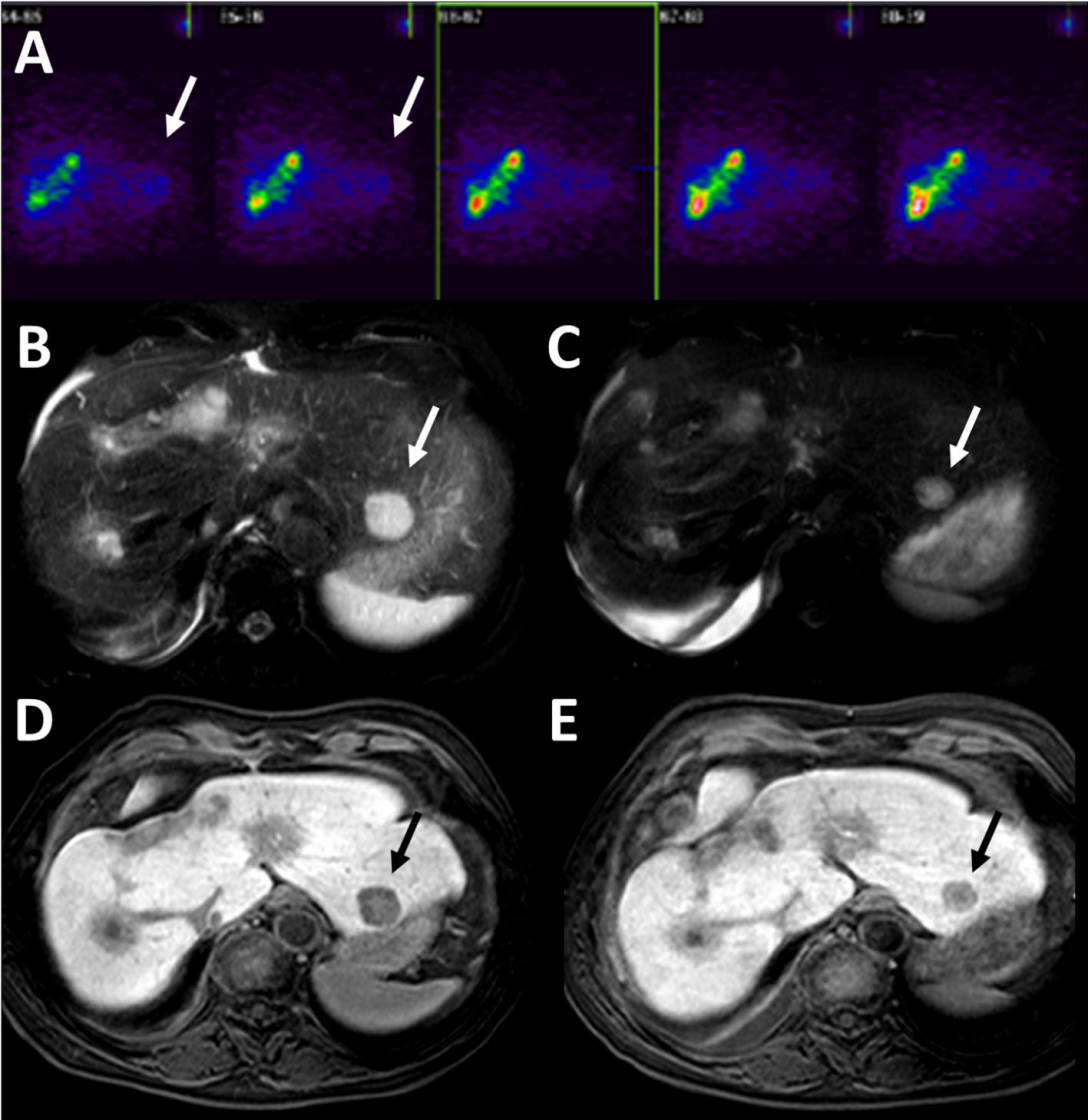
Patient B, prostate cancer, A-E, questionable abscopal effect: **A** SPECT (without CT): retrospectively uncertain whether there is some ^90^Y contamination on the left side (white arrow). **B** and **C** MRI1 and MRI2: Metastasis size regression (white arrow) in fat saturated T2w MRI. **D** and **E** MRI1 and MRI2: black arrow shows size regression in T1w 20 min after administration of primovist

One certain AE was registered in patient A (Fig. 2, Table 2). Another patient (patient B) had a questionable AE (Fig. 3, Table 2). The reason why this AE remains questionable is potential partial contamination, based on the appearance of the Bremsstrahlung imaging, of the left liver lobe with some activity during the RE of the right liver lobe.

**Fig. 4:**
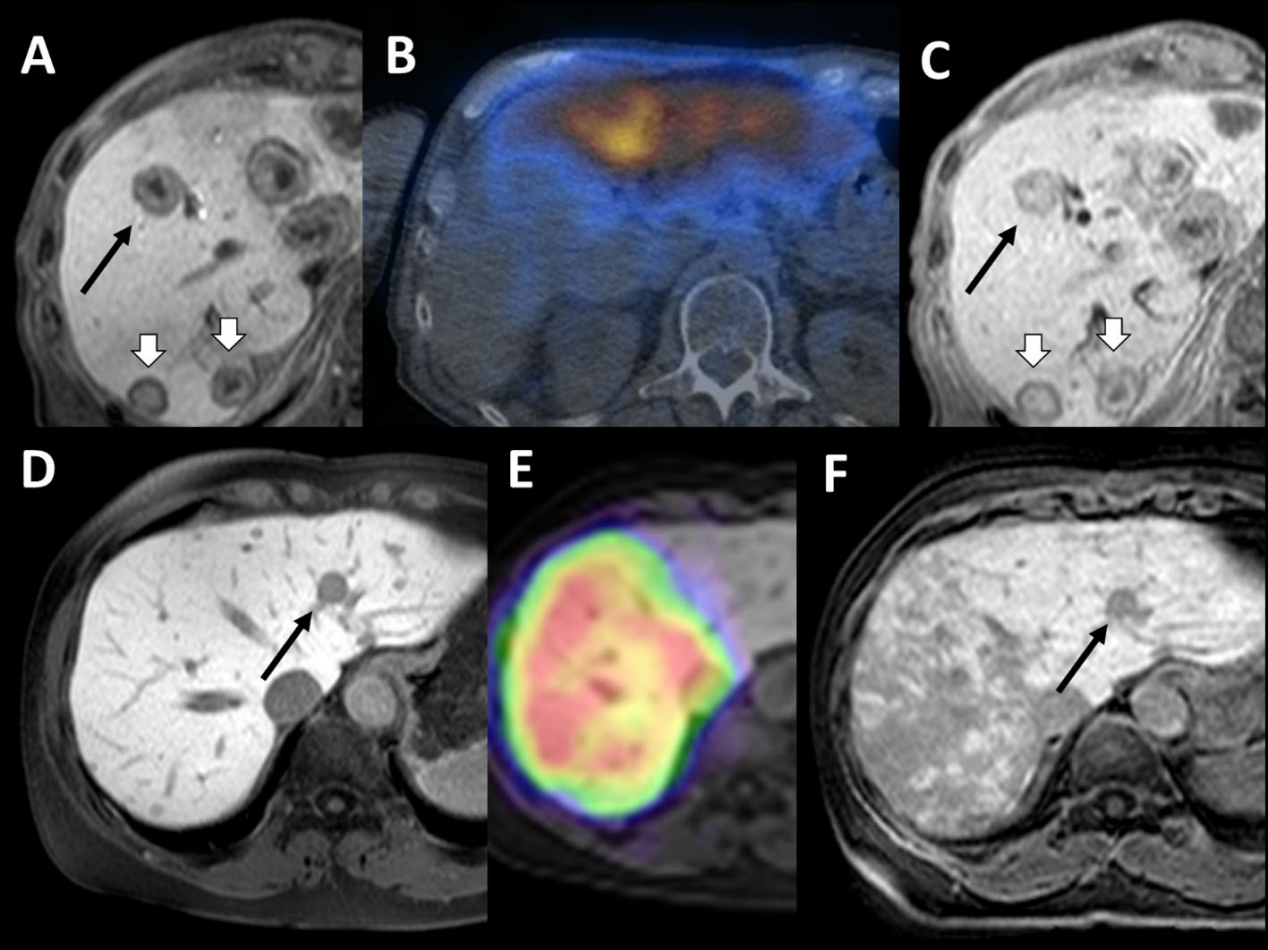
Two cases of size regression < 30%, but noticeably smaller, **A**–**C** nasopharyngeal carcinoma,** D**–**F** breast cancer: **A** and **C**) black arrow show a metastasis decreasing from 22 to 20 mm (i.e., 10%), white arrows indicate progressing lesions, e.g., 18–20 mm and 19–22 mm.** B** Bremsstrahlung-SPECT/CT examination: RE left and segment 4b. Untreated segments 5–8 and 4a. **D** and **E** black arrow: 12–10 mm (~ 20%). **F** Radiation damage in the liver, typical RE image for breast cancer

Furthermore, the analysis revealed two patients whose untreated metastases demonstrated noticeable shrinkage but < 20% in diameter (Fig. 4). One of these two patients had a nasopharyngeal carcinoma (Fig. 4, A-C), the other had breast cancer (Fig. 4, D-F).

## Discussion

Despite the growing understanding of AE following radiotherapy and its underlying immune-mediated-mechanisms, its clinical incidence remains low and its triggers undeciphered. AE exist at all ages, across a variety of tumor entities and with substantial differences in radiotherapy regimens and techniques. According to a recent review, 46 case reports exist concerning AE from radiotherapy (1969–2014) [14]. Most reported cases of AE occur in immunogenic tumors like melanoma with high mutation burden. Immunogenicity of tumors can be modulated by high linear energy transfer (LET), which applies a high-density ionization, leading to a release of neoantigens due to its lesser dependency on tissue oxygenation and direct DNA damage demonstrating high biological impact [15]. The heterogenous BC includes several different histological and four molecular subtypes, which have significantly different immunogenic properties [16]. Correspondingly, γ-irradiation induces different forms of cell death in BC depending on the genetic thumbprint and the radiation regimen [17]. In triple-negative BC, the highest concentration of DAMPs was found after high-single-dose-irradiation at 20 Gy resulting in a late onset primary necrosis with features of mitotic catastrophe and plasma membrane disintegration—especially four days after irradiation [17].

In our study, four patients demonstrated tumor-regression distant to the site treated with RE. One patient with breast cancer (patient A) showed the most noticeable tumor shrinkage. Breast cancer (BC) has generally been considered a poorly immunogenic tumor type [16, 18]. The molecular subtype has inherent clinical relevance in terms of therapy (e.g., hormone receptor inhibitors) and both quantity and composition of tumor infiltrating leukocytes (12). The resulting tumor microenvironments probably affect any AE as well, to which extent, however, remains speculative at this point. The current evidence mostly suggests a significant correlation between immune cells and clinical outcome for estrogen receptor negative breast cancer, which does not apply to our patient (PR + , ER + , HER2−, Ki67 unknown ) (12).

The other patient (patient B) demonstrating an AE, which might however be attributed to possible Y-90 contamination during RE, had prostate cancer, a tumor entity known for its highly immunosuppressive microenvironment and low mutation burden [19].

Optimal radiation doses and fractionation regimes to induce AE with radiotherapy are still discussed controversially, and a consensus was not found up to this point, especially in combination with various immunotherapeutic options [20]. Very few researchers compare different irradiation regimes in their studies about AE. Published results are conflicting, prone to misunderstandings and difficult to compare with inconsistent usage of terminology. However, most published studies concerning this topic lean towards high-dose, hypofractionated regimes. Animal models indicate a fivefold higher AE incidence with high-dose, hypofractionated radiation regimes compared to the traditional (hyper-)fractionated approach [8, 21]. Radioembolization with 90-Y microspheres employs high estimated tumor doses with an average of 200-300 Gy up to a maximum of 3000 Gy [22], making it a potential initiator of any AE in theory, ideally catalyzed by immunotherapeutic agents. The best RT regimes to induce an AE are unknown. Growing consensus indicates that an optimal radiation dose-range likely exists, below which immune stimulation might be inefficient and above which immunosuppression predominates [23]. The ideal RE dose to initiate a sufficient or even optimal immune stimulation could be difficult to calculate, because the applied activity corresponds to liver and tumor volumes. Experience concerning RE and AE remains scarce. To our knowledge, there are only three case reports—two of which combine radio immunotherapy [12, 24, 25].

Tumor volume or metastasis mass usually leads to a different applied radiotherapeutic regime when comparing the clinical routine and the case reports of radiation-induced AE. On the one hand, lesions might be pre-treated in the clinical context—diminishing any out-of-field response; on the other hand, it was demonstrated that systemic anti-tumor immunity declines if the tumor size exceeds a certain threshold. Evolving tumors generally establish a highly immunosuppressive microenvironment infiltrated by regulatory-T-cells (Tregs), myeloid-derived suppressor cells and alternatively activated macrophages [26]. The strength of inducible anti-tumor immunity most likely depends on the tumor volume.

Radioembolization presents a rather second-tier anti-neoplastic treatment option usually applied as a salvage therapy in a palliative setting. The increasing interest in AE could promote the wider RE application and enhance its status to a rather primary tool of the anti-neoplastic toolbox when combined with immunotherapeutic agents like anti-CTLA-4, PD-1 antagonists, GM-CSF or IL-2. RE can effectively present tumor antigens and initiate T-cell extravasation; however, RE alone accomplishes little to overcome the suppressive tumor microenvironments. One retrospective study examined the safety of combination RE and checkpoint inhibitor immunotherapy; limited toxicity was observed and the importance to find an ideal combination protocol was emphasized [27]. The regulation of AE relies on a delicate balance between immune suppression and immune activation. AE is immune mediated: radiation induces local inflammation and augments T-cell activation ultimately leading to cancer cell elimination. The rarity of AE is attributed to the highly immunosuppressive environment of many tumor entities, resulting in increasing efforts to combine radiotherapy and immunomodulating drugs to override tumor immune evasion. The ideal time to administer immunotherapeutic agents in combination with radiotherapy remains unknown, hence the quite arbitrary application time at this point—either before, concurrent or after radiotherapy. The occurrence of AE was demonstrated to be increased by simultaneous/sequential immunotherapy [9]. Both single and fractionated radiation regimens were reported to boost AE combined with different immunotherapies [28–30]. Some of the most promising immunotherapies for AE are anti-CTLA4 (Ipilimumab), PD1/ PD-L1 (Nivolumab) and GM-CSF as indicated in several studies [9]. Ipilimumab, the most frequently used substance, has been demonstrated to enhance the incidence of AE to 25% in melanoma patients [10]. However, Ipilimumab leads to a broad systemic activation of T-cells and consequently powerful immune cell infiltration but also result severe potential side effects [31]. Another restriction is its limited tissue penetration capability [32]. T-cell activation by anti-PD-1/PD-L1 seems to be subtler. According to several studies, GM-CSF can increase the incidence of AE to 30% and programmed-cell-death-ligand-1 (PDL1) antibodies to 25% [9, 11]. In our retrospective study, the observed AE rate without additional immunotherapy is less than 10%. The targeted nature of Yttrium-90 might be suitable for the installation of immunotherapeutic agents and to initiate an antigenic cascade ultimately resulting in AE. Many ongoing studies are currently investigating the combination of radiotherapy in general and immunotherapy to boost AE incidences and improve treatment outcomes for various indications [33]. Preclinial trials suggest that dosage, timing and combinations are crucial when hoping for success of combined radio immunotherapy. Low immunogenicity of tumor antigens at the local site of irradiation as well as immunosuppressive cells and cytokines limit the AE even in combination approaches. Most reported cases of AE occur in immunogenic tumors [34]. In general, any factors that suppress the patient’s immune system may prevent the development of AE as well as other factors like myelosuppression, overall tumor burden, lymphocyte ratio or prior exposure to radiation or cytotoxic chemotherapy [33]. In our study, patients had to discontinue their prior treatment at least four weeks before RE—still any hampering effects of prior systemic treatments are possible.

The study has several limitations. The time to observe/expect an AE after radiotherapy and possibly immunotherapy remains unknown. A recent review of 46 AE case reports calculated the median time of 2 months (range 0–24 months) to document an AE. In our study, we chose a relatively short timeframe with a median of 34 days (20–54) after RE to search for AE in the follow-up MRI. This was done to be certain that any potential AE effect was not caused by some other form of therapy (e.g., chemotherapy) and due to the sequential setting of RE (untreated liver lobe was scheduled for RE several weeks later). Although the ideal timeframe to expect an out-of-field effect after radiotherapy remains unknown, it appears rational to assume that we missed several cases of AE because of the prompt follow-up in our study. Furthermore, this study evaluates only intra-hepatic and no extrahepatic AE. Systemic AE was not looked for mainly because the included patients only receive a liver MRI as an immediate short term imaging follow-up (whole-body CT was acquired much later).

Major issues, e.g., finding an optimal timeframe and radiotherapy regimen, must be solved to increase the incidence of AE, unlock its true potential and raise its clinical importance to improve the outcome of various cancer entities. Prospective trials combining different immunotherapeutic agents and radioembolization are underway to induce a more efficient immune response.

In conclusion, although AE is very rare and only one certain case was identified in this study of immunotherapy naïve patients, there is sufficient incentive to further examine the induction of this phenomenon. The synergy of radiotherapy/immunotherapy potentially offers the opportunity to generate some form of in situ cancer vaccine, which would then provide cancer “immunity” for some time.
